# Rehabilitation of a Maxillary Defect and Missing Teeth in the Esthetic Zone With a Long‐Span Fixed Dental Prosthesis: A Case Report

**DOI:** 10.1155/crid/7652084

**Published:** 2026-10-09

**Authors:** Aleksandra Prokić, Stefan Vulović, David Krcunović, Miloš Pelemiš, Aleksandra Milić Lemić

**Affiliations:** ^1^ Department of Prosthodontics School of Dental Medicine University of Belgrade Belgrade Serbia bg.ac.rs; ^2^ Dental Lab Dk_smilecreator Belgrade Serbia

**Keywords:** case report, esthetic zone, fixed dental prosthesis, maxillary defect

## Abstract

**Background:**

Rehabilitation of the esthetic zone is one of the greatest challenges in contemporary prosthodontics. In addition to tooth loss, defects of the alveolar ridge and surrounding soft tissues often compromise functional and esthetic outcomes. When implant therapy is contraindicated or not feasible, treatment should be tailored to the patient′s individual needs. The aim of this case report was to present the prosthetic rehabilitation of a complex maxillary defect and missing teeth in the esthetic zone using a long‐span fixed dental prosthesis (FDP).

**Case Report:**

A 51‐year‐old female patient was referred to the Department of Prosthodontics, School of Dental Medicine, University of Belgrade, for prosthetic rehabilitation following surgical enucleation of a large cystic lesion in the anterior maxilla. The procedure required extraction of the maxillary incisors and canines, resulting in extensive hard and soft tissue loss in the esthetic zone. The maxillary premolars and first molars were prepared as abutments, and a provisional polymethyl methacrylate (PMMA) bridge was fabricated. Three weeks later, a definitive porcelain‐fused‐to‐metal FDP was manufactured. The metal framework and ceramic try‐ins were used to evaluate fit, oral hygiene accessibility, occlusion, esthetics, and shade before fabrication of the definitive restoration. After glazing, the long‐span FDP was cemented. At the 1‐year follow‐up, the final restoration demonstrated satisfactory function, esthetics, and stability.

**Conclusion:**

Long‐span porcelain‐fused‐to‐metal FDPs can provide a predictable treatment option for selected patients with a maxillary defect and missing teeth in the esthetic zone when implant therapy is not indicated. Careful treatment planning and individualized prosthetic rehabilitation are essential for achieving favorable functional and esthetic outcomes. Further clinical studies with larger samples and longer follow‐up are needed to evaluate the long‐term success of this treatment approach.

## 1. Introduction

Rehabilitation of the esthetic zone remains one of the most demanding challenges in contemporary prosthodontics. Replacing missing maxillary anterior teeth and the associated hard and soft tissues requires clinicians to simultaneously restore function, phonetics, biological health, and natural esthetics. Achieving an optimal outcome depends on careful evaluation of multiple factors, including the smile line, lip support, gingival morphology, anterior guidance, occlusal relationships, and the morphology, color, and position of the restoration. Because even minor discrepancies in this region are readily perceived by both patients and clinicians, treatment planning must be highly individualized and based on a comprehensive clinical and radiographic assessment.

The maxillary anterior teeth play a fundamental role in facial appearance, speech, mastication, and psychosocial well‐being. Previous studies have demonstrated that dissatisfaction with dental appearance is strongly associated with reduced self‐esteem, impaired social interactions, and decreased oral health–related quality of life (OHRQoL) [1]. Consequently, successful rehabilitation of the anterior teeth in the maxilla extends beyond replacing missing teeth, as it also aims to restore patients′ confidence, comfort, and overall quality of life. Establishing a harmonious relationship between the restoration, surrounding soft tissues, and facial characteristics is therefore essential for achieving long‐term success.

Maxillary defects may result from congenital or developmental abnormalities, trauma, periodontal disease, tumor resection, infection, or previous surgical interventions [2]. The severity of these defects varies considerably and may involve the loss of teeth, alveolar bone, and soft tissues, making rehabilitation increasingly complex. Careful diagnosis, interdisciplinary treatment planning, and an accurate evaluation of the remaining dentition, periodontal support, bone volume, and patient expectations are crucial for selecting the most appropriate treatment modality. Depending on the clinical situation, treatment options include implant‐supported fixed dental prostheses (FDPs) combined with bone augmentation procedures, conventional FDPs, implant‐assisted removable prostheses, combinations of fixed and removable partial dentures (RPDs), or conventional removable prostheses.

Although implant‐supported restorations are widely considered the preferred treatment option for replacing missing teeth in many clinical situations, they are not universally applicable. Some patients decline implant therapy because of the need for surgical intervention, treatment duration, financial considerations, or personal preferences. In addition, severe alveolar bone loss, extensive hard and soft tissue defects, systemic medical conditions, or other local and general contraindications may preclude implant placement. Under these circumstances, conventional FDPs remain a predictable and effective treatment alternative when appropriately planned and executed.

Depending on the edentulous span, FDPs are generally classified as short‐span prostheses, replacing three or four units, or long‐span prostheses, replacing five or more units. Whenever possible, short‐span FDPs are preferred because of their more favorable biomechanical behavior and prognosis. However, long‐span FDPs may represent the most appropriate treatment option in carefully selected patients presenting with extensive anterior defects, provided that adequate abutment teeth, occlusal conditions, and prosthetic principles are respected. Despite their increased biomechanical complexity, advances in restorative materials, adhesive techniques, and prosthetic protocols have expanded the indications for long‐span fixed restorations.

The aim of this case report was to present the prosthetic rehabilitation of a complex maxillary anterior defect and missing teeth using a long‐span FDP. The report describes the diagnostic process, treatment planning, clinical workflow, and prosthetic management, highlighting the functional and esthetic outcomes achieved in a patient for whom implant therapy was not considered a suitable treatment option.

## 2. Case Presentation

A 51‐year‐old female patient was referred to the Department of Prosthodontics, School of Dental Medicine, University of Belgrade, in February 2025 seeking prosthetic rehabilitation following extensive tooth loss in the anterior maxilla. On October 1, 2024, the patient had undergone surgical enucleation of a large cystic lesion involving the anterior maxillary region, which necessitated the extraction of both maxillary central incisors, lateral incisors, and canines. Histopathological examination subsequently confirmed the diagnosis of a radicular cyst. The resulting defect involved not only the loss of teeth but also a significant deficiency of the supporting hard and soft tissues (Figure 1), creating a considerable functional and esthetic challenge. During the interval between the surgical procedure and the referral to the Department of Prosthodontics, the patient did not use any fixed or removable provisional prosthesis.

**Figure 1:**
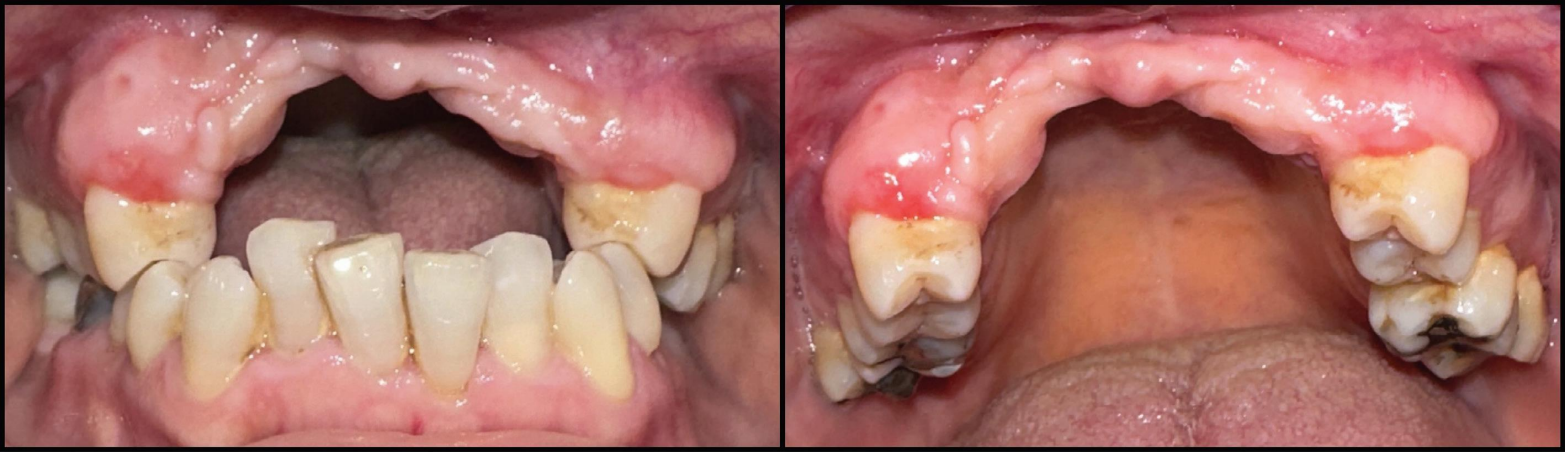
Patient with extensive tooth loss and significant deficiency of the supporting hard and soft tissues in the anterior maxilla.

The patient denied any systemic diseases, regular use of prescription or over‐the‐counter medications, and any known allergies to medications or food. The patient was a smoker, reporting a consumption of fewer than 10 cigarettes per day, and denied alcohol consumption. Her family history was noncontributory, with no similar lesions or related conditions reported among first‐degree relatives. The cystic lesion was detected incidentally during routine radiographic follow‐up (Figure 2), as the patient was completely asymptomatic and reported no pain, swelling, sensory disturbances, or any other clinical signs suggestive of its presence.

**Figure 2:**
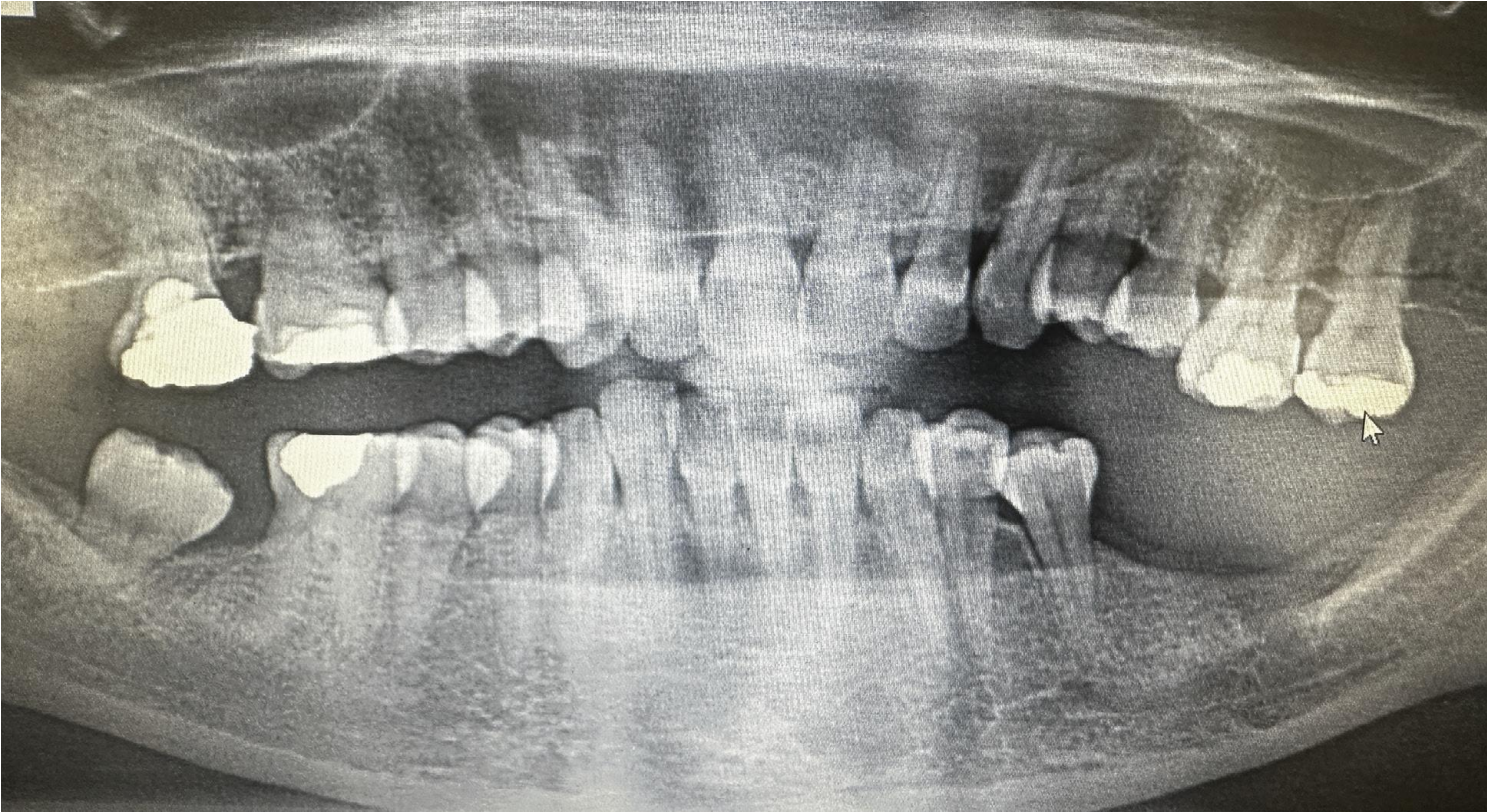
Panoramic radiograph (orthopantomogram) demonstrating the presence of a cystic lesion in the anterior region of the maxilla.

A comprehensive clinical and radiographic examination was performed. Based on Seibert′s classification of residual ridge deformities [3], the defect was diagnosed as a severe Class III ridge defect, characterized by combined horizontal and vertical loss of the alveolar ridge. The extent of the defect significantly compromised the available prosthetic support and limited the treatment options. Several treatment alternatives were discussed with the patient, including an implant‐supported FDP following extensive autogenous bone grafting, a conventional tooth‐supported FDP, a combined fixed‐removable prosthesis, and a conventional RPD.

Although implant‐supported rehabilitation represents the preferred treatment modality in many cases of anterior tooth loss, the severity of the hard and soft tissue deficiency would have required extensive reconstructive surgery with an uncertain long‐term prognosis and prolonged treatment time. Furthermore, the patient expressed a strong preference for a fixed prosthetic solution and declined removable treatment options. Considering the remaining dentition, biomechanical requirements, and the patient′s expectations, rehabilitation with a long‐span tooth‐supported porcelain‐fused‐to‐metal FDP was selected as the definitive treatment.

Prior to treatment, OHRQoL was evaluated using the Serbian version of the Oral Health Impact Profile‐5 (OHIP‐5‐Srb) (Table 1). The questionnaire was translated and culturally adapted from the original English version in accordance with established translation guidelines [4]. The OHIP‐5‐Srb consists of five questions assessing key aspects of OHRQoL, including difficulties with chewing, oral pain, dissatisfaction with dental or facial appearance, reduced enjoyment of food due to oral problems, and limitations in performing usual daily activities caused by conditions affecting the teeth, mouth, dentures, or jaws. Each item was assessed using a 5‐point Likert scale with descriptive response options only (*never*, *hardly ever*, *occasionally*, *fairly often*, and *very often*) rather than numerical scores (0–4), as the questionnaire was administered to a single patient.

**Table 1:** Oral health impact profile (OHIP)‐5‐Srb questionnaire used to evaluate oral health–related quality of life (OHRQoL).

	Oral health impact profile (OHIP)‐5‐Srb			
	Initial	Provisional restoration 3 weeks follow‐up	Definitive restoration 3 weeks follow‐up	Definitive restoration 1 year follow‐up
Difficulties with chewing	Very often	Occasionally	Occasionally	Hardly ever
Oral pain	Never	Never	Never	Never
Dissatisfaction with dental or facial appearance	Very often	Never	Never	Never
Reduced enjoyment of food due to oral problems	Very often	Occasionally	Occasionally	Hardly ever
Limitations in performing usual daily activities caused by conditions affecting the teeth, mouth, dentures, or jaws	Never	Never	Never	Never

In addition to the OHIP‐5‐Srb questionnaire, the plaque index (PI) was assessed before treatment to determine the presence of dental plaque on the surfaces of the abutment teeth selected for prosthetic restoration (Table 2). Plaque accumulation was evaluated at six predefined sites per tooth (three vestibular and three palatal) using a periodontal probe (PCPUNC‐15, HU‐Friedy, Chicago, Illinois, United States) [5]. Each site was scored as 0 if no plaque was detected and 1 if plaque was present, whereas PI was calculated according to the following formula:

PI%=Plaque positive sites/total number of examined sites×100.

**Table 2:** Plaque index (PI) used to determine the presence of dental plaque on the surfaces of the abutment teeth selected for provisional/definitive restoration.

Plaque index (PI)			
Initial	Provisional restoration 3 weeks follow‐up	Definitive restoration 3 weeks follow‐up	Definitive restoration 1 year follow‐up
13/24 surfaces (54.17%)	11/24 surfaces (45.84%)	14/36 surfaces (38.89%)	13/36 surfaces (36.11%)

At the initial examination, before treatment, the patient experienced difficulties with chewing, reduced enjoyment of food due to oral problems, and dissatisfaction with dental or facial appearance very often (Table 1). In contrast, no oral pain was reported, and the patient did not experience limitations in performing usual daily activities related to oral conditions. The baseline PI (Table 2) showed plaque accumulation on 13 out of 24 examined surfaces (54.17%) (four abutment teeth for provisional restoration × six surfaces), indicating a moderate level of plaque presence around the teeth planned to serve as abutments for the provisional restoration.

The initial clinical phase consisted prosthodontic preparation of the maxillary first and second premolars, which were planned as the abutment teeth for provisional restoration. Standard tooth preparation for full‐coverage porcelain‐fused‐to‐metal crowns was performed, ensuring adequate reduction for restorative material thickness while preserving as much sound tooth structure as possible.

A 10‐unit provisional FDP was designed in computer software (DentalCAD 3.2 Elefsina, Exocad, Darmstadt, Germany), then fabricated from polymethyl methacrylate (PMMA) disc (Telio CAD, Ivoclar Vivadent, Schaan, Liechtenstein) in a computer‐controlled machine (XTCERA 500 Plus, Shenzhen Xiangtong, Shenzhen, Guangdong, PR China) under constant water‐cooling. The finishing and polishing procedure was performed following manufacturer recommendations.

The provisional restoration served several important purposes throughout the treatment. It restored esthetics and function, allowed evaluation of phonetics and occlusal relationships, facilitated conditioning of the surrounding soft tissues and remodeling of the residual alveolar ridge, and enabled assessment of the patient′s ability to maintain adequate oral hygiene. Furthermore, it provided valuable insight into the patient′s adaptation to the proposed definitive restoration and served as a guide for the dental technician in fabricating a predictable and well‐fitting definitive restoration (Figure 3).

**Figure 3:**
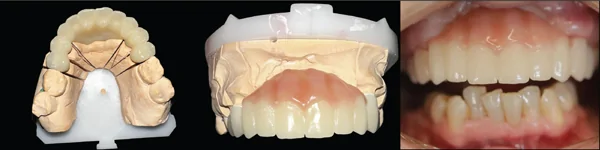
Provisional PMMA fixed dental prosthesis.

The provisional FDP was worn for approximately 2 months, from February 2025 until the fabrication of definitive restoration in April 2025. This period allowed for further soft‐tissue maturation and residual ridge remodeling, as well as evaluation of phonetics, occlusal relationships, oral hygiene accessibility, and the adaptation of the pontics to the residual ridge prior to fabrication of the definitive restoration. Three weeks after delivery of the provisional restoration, an improvement in patient‐reported outcomes was observed (Table 1). Chewing difficulties decreased from very often to occasionally, and dissatisfaction with dental appearance was completely resolved, changing from very often at baseline to never after placement of the provisional restoration. Reduced enjoyment of food due to oral problems also improved, decreasing from very often to occasionally. No oral pain or limitations in daily activities were reported. The PI demonstrated a slight reduction in plaque accumulation (Table 2), with 11 out of 24 examined surfaces affected (45.84%), suggesting slightly improved oral hygiene conditions during the provisional restoration phase.

Clinical and occlusal examination revealed no clinically significant extrusion of the mandibular incisors. Nevertheless, their position and occlusal relationship with the planned maxillary FDP were carefully considered during treatment planning. The occlusal scheme was initially evaluated and refined using the provisional restoration. The palatal surfaces of the maxillary anterior units were designed to establish appropriately distributed anterior contacts and provide smooth guidance during protrusive and other eccentric mandibular movements, while avoiding premature contacts and occlusal interferences. Comprehensive rehabilitation of the mandibular arch was planned as a subsequent treatment phase to increase the number of posterior occlusal contacts, optimize the distribution of occlusal forces, and potentially reduce the mechanical load on the long‐span FDP.

Considering the length of the definitive restoration and the anticipated functional loading, the treatment plan was modified to include both maxillary first molars as additional abutment teeth. These teeth were also prepared following standard tooth preparation for full‐coverage porcelain‐fused‐to‐metal crowns. The maxillary right second molar was considered to have an unfavorable long‐term prognosis and was therefore intentionally excluded from the definitive FDP, with the first molar serving as the terminal abutment on the right side. Once all abutment teeth had been prepared (Figure 4), the definitive impression was obtained using a two‐step putty‐wash technique with polyvinyl siloxane impression material. A putty material (Elite HD+ Putty Soft, Zhermack, Badia Polesine, Italy) was used for the preliminary impression, followed by a light‐body material (Elite HD+ Light Body, Zhermack) to accurately capture the finish lines and surrounding soft tissues, followed by fabrication of the definitive cast.

**Figure 4:**
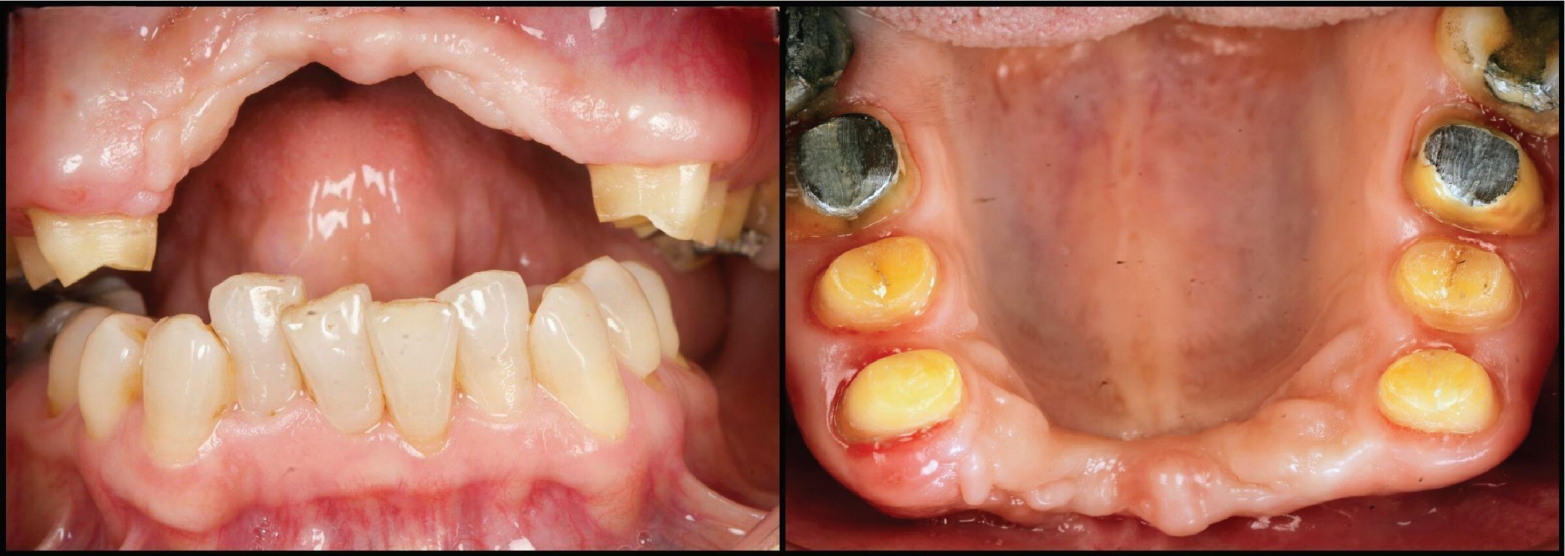
Prepared teeth for fabrication of definitive restoration.

Special consideration was given to minimizing the weight of the long‐span restoration due to the extensive pontic segment replacing the lost alveolar ridge and anterior teeth. Therefore, the metal framework was designed with a hollow internal structure while maintaining adequate rigidity and support for the veneering ceramic. The framework was fabricated from CoCr alloy using a selective laser melting (SLM) metal additive manufacturing system (Riton MLAB, Riton Biomaterial, Shanghai, PR China). The printing parameters were set as follows: a layer thickness of 30 *μ*m, laser power of 200 W, laser scanning speed of 800 mm/s, hatch spacing of 0.08 mm, and a stripe scanning strategy with a rotation angle of 67° between successive layers.

The build orientation was set according to the manufacturer′s recommendations to optimize accuracy and minimize deformation during fabrication. After printing, the metal framework was removed from the build platform, and residual powder was eliminated using compressed air and ultrasonic cleaning. The printed frameworks were subsequently subjected to postprocessing according to the manufacturer′s protocol, including heat treatment for stress relief and surface finishing prior to ceramic veneering. The initial printed framework weighed 16.0 g. Following finishing and surface processing, approximately 1.7 g of material was removed, resulting in a final framework weight of 14.3 g before ceramic application. The framework was clinically evaluated to verify passive fit, marginal adaptation, stability, and available restorative space (Figure 5).

**Figure 5:**
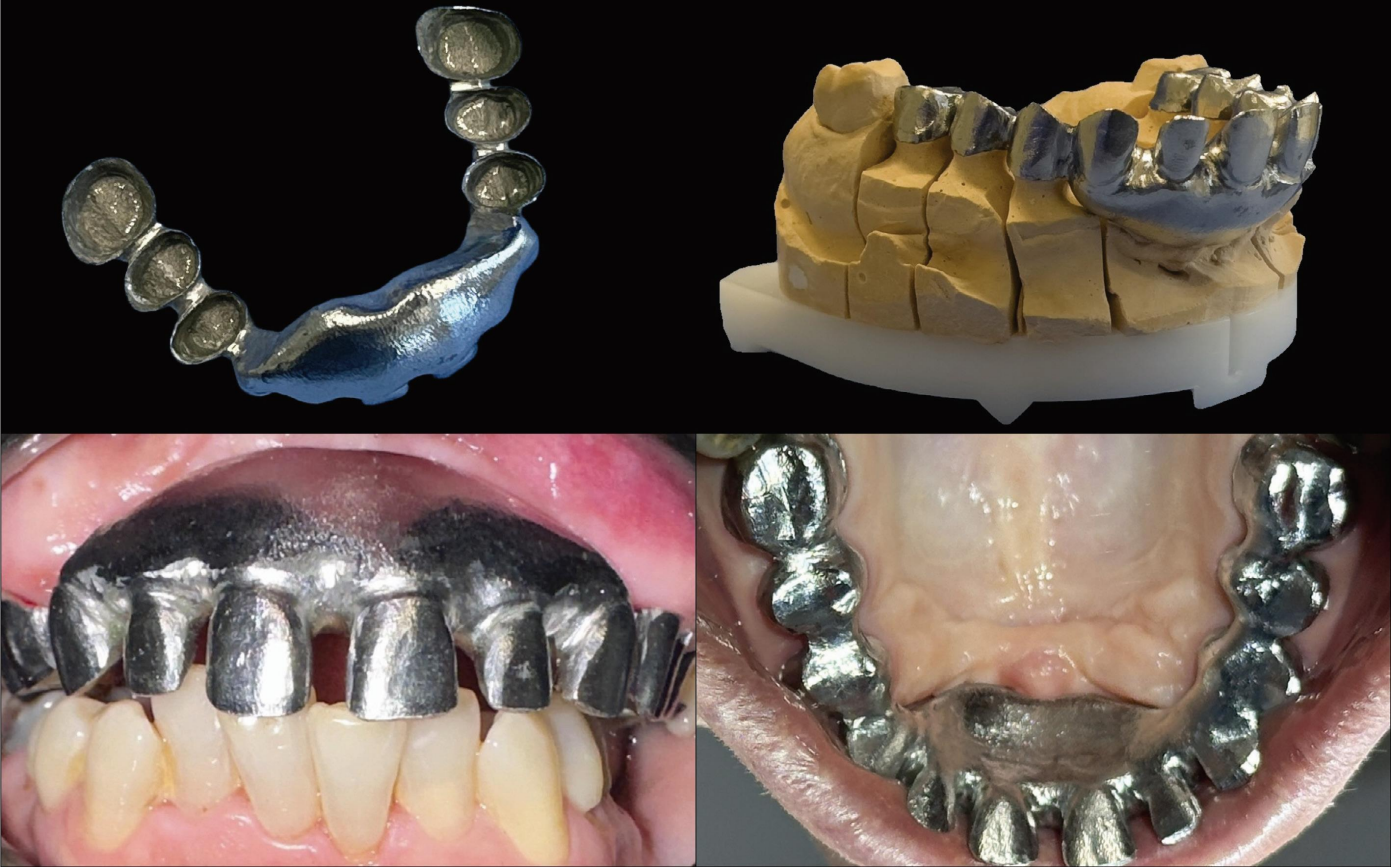
Three‐dimensional (3D) printed CoCr alloy metal framework and its clinical evaluation during intraoral assessment.

Following successful verification, veneering porcelain (IPS Style, Ivoclar Vivadent) was layered onto the metal framework according to the manufacturer′s recommendations. The completed restoration weighed 19.8 g, demonstrating that the hollow framework design substantially reduced the overall weight of the restoration while maintaining adequate structural integrity. The definitive restoration replaced the missing teeth as well as the lost alveolar contour, providing satisfactory lip support and significantly improving facial esthetics (Figure 6). Prior to cementation, the restoration was evaluated for marginal adaptation, occlusal contacts, centric and eccentric mandibular movements, phonetics, esthetics, and accessibility for oral hygiene procedures. Minor occlusal adjustments were performed where necessary.

**Figure 6:**
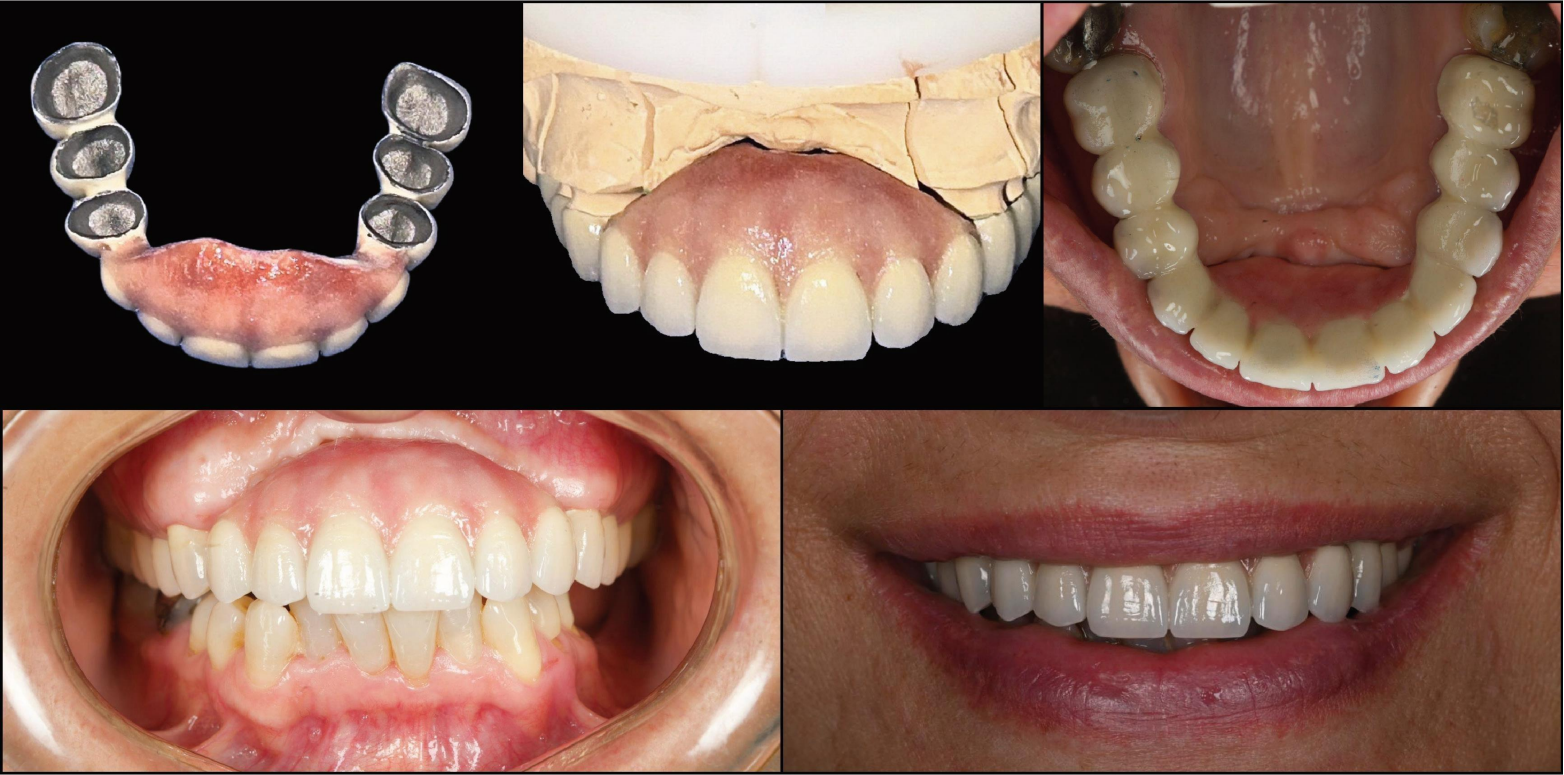
Definitive porcelain‐fused‐to‐metal fixed dental prosthesis restoring the missing teeth and the lost alveolar contour.

The definitive porcelain‐fused‐to‐metal FDP was permanently cemented using a resin‐modified glass ionomer cement (GC Fuji PLUS, GC, Tokyo, Japan). Postoperative oral hygiene instructions were thoroughly explained, with particular emphasis on cleaning beneath the pontic area using super floss and interdental cleaning aids. The patient was enrolled in a regular maintenance program with scheduled follow‐up appointments to monitor oral hygiene, periodontal health, prosthesis integrity, and long‐term clinical performance.

Three weeks after delivery of the definitive restoration, the patient continued to report improved OHRQoL (Table 1). Chewing difficulties and reduced enjoyment of food remained present only occasionally, whereas dissatisfaction with dental or facial appearance remained absent (never). Oral pain and functional limitations were also not reported. The PI was calculated on 14 out of 36 examined surfaces (38.89%) (Table 2), showing a further reduction in the proportion of plaque‐positive sites compared with the initial examination and the provisional restoration phase. These findings indicate a positive clinical and patient‐perceived response following placement of the definitive restoration.

At the 1‐year follow‐up after delivery of the definitive restoration (Figures 7 and 8), the patient demonstrated further improvement in perceived oral health status (Table 1). Chewing difficulties decreased to hardly ever, whereas reduced enjoyment of food due to oral problems also decreased to hardly ever, indicating sustained functional improvement over time. The patient continued to report no oral pain, dissatisfaction with dental appearance, or limitations in daily activities. The PI remained stable (Table 2), with plaque detected on 13 out of 36 examined surfaces (36.11%), representing the lowest plaque accumulation recorded during the observation period. Overall, the 1‐year evaluation demonstrated maintained clinical conditions, improved oral hygiene status, and a sustained positive impact of the prosthetic rehabilitation on OHRQoL.

**Figure 7:**
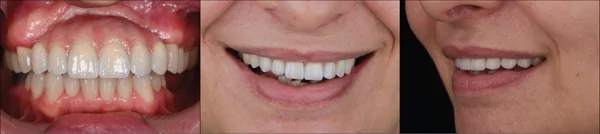
Definitive porcelain‐fused‐to‐metal fixed dental prosthesis at 1‐year follow‐up after delivery of the definitive restoration.

**Figure 8:**
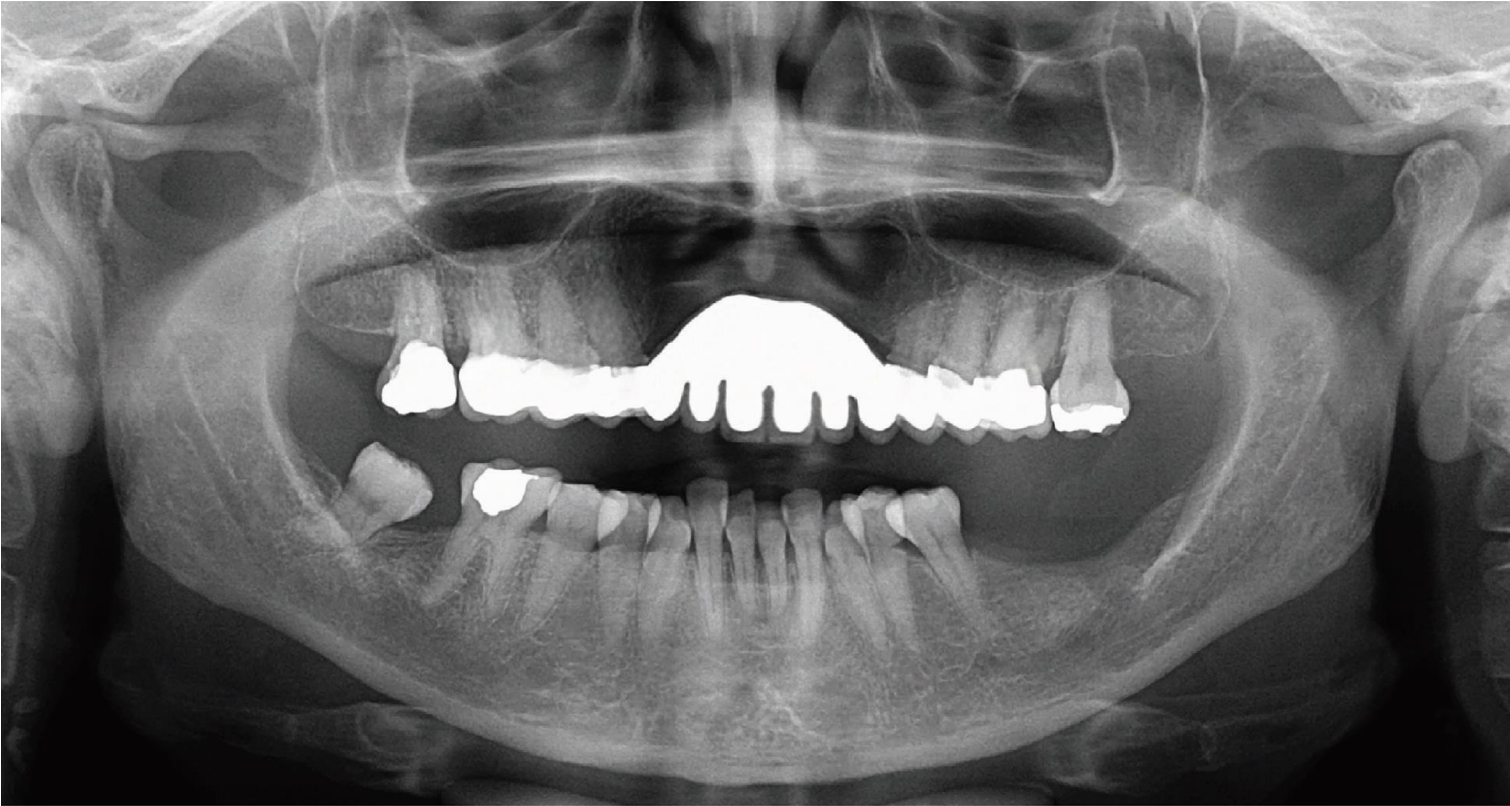
Panoramic radiograph (orthopantomogram) at 1‐year follow‐up after delivery of the definitive restoration.

## 3. Discussion

The loss of anterior maxillary teeth accompanied by extensive hard and soft tissue destruction represents one of the most demanding situations in prosthodontic rehabilitation. Surgical treatment of cystic lesions frequently results in significant alveolar ridge resorption, further compromising prosthetic treatment. According to Seibert′s classification [3], alveolar ridge defects are categorized into Class I (horizontal loss), Class II (vertical loss), and Class III (combined horizontal and vertical loss). The present patient exhibited a severe Seibert Class III defect, characterized by both vertical and horizontal ridge deficiency, making prosthetic rehabilitation particularly challenging from functional, biomechanical, and esthetic perspectives. Residual ridge resorption is a progressive and irreversible process that continues after tooth extraction, particularly in patients who do not receive timely prosthetic rehabilitation or implant therapy [6, 7]. Severe ridge defects not only compromise prosthesis support but also affect lip support, facial appearance, phonetics, and oral function. Consequently, treatment planning should be individualized, taking into consideration the extent of tissue loss, remaining dentition, systemic conditions, patient expectations, and long‐term prognosis.

Beyond the functional consequences, tooth loss has a profound psychosocial impact. Numerous studies have demonstrated an association between poor oral health, reduced number of remaining teeth, and increased prevalence of anxiety, depression, impaired sleep quality, and reduced quality of life [8–13]. The loss of anterior teeth is particularly distressing because of its immediate influence on facial esthetics and speech, frequently resulting in decreased self‐confidence and social withdrawal [14, 15]. Therefore, successful rehabilitation should address not only restoration of masticatory function but also the patient′s psychological well‐being and overall quality of life.

The combination of the OHIP‐5‐Srb questionnaire and the PI provided a comprehensive assessment of both the patient′s subjective perception of oral health and the objective clinical status of oral hygiene. The OHIP‐5‐Srb is a validated, short, and patient‐centered instrument designed to evaluate the impact of oral conditions on daily life, including functional limitations, discomfort, esthetic concerns, and psychosocial aspects. Because of its simplicity and low respondent burden, it is particularly suitable for longitudinal monitoring of changes in OHRQoL during different phases of prosthetic treatment [16]. Although longer OHIP versions (OHIP‐14 and OHIP‐49) assess four domains, such as oral function, orofacial pain, orofacial appearance, and psychosocial impact, the OHIP‐5 has consistently demonstrated a unidimensional structure across validation studies [17–19]. Nevertheless, its five items represent all major aspects of OHRQoL: chewing difficulty (oral function), painful aching (orofacial pain), discomfort about appearance (orofacial appearance), and reduced food flavor and difficulty performing usual activities (psychosocial impact).

The PI complements the subjective assessment by providing an objective measure of dental plaque accumulation, which is closely associated with oral hygiene status and the long‐term maintenance of periodontal and prosthetic health. Regular evaluation of plaque levels around abutment teeth is especially important in fixed prosthetic rehabilitation, as inadequate plaque control may contribute to periodontal inflammation and compromise the longevity of the restoration. Together, OHIP‐5‐Srb and PI allow simultaneous evaluation of the patient′s perceived treatment outcomes and clinically measurable oral conditions. While OHIP‐5‐Srb reflects the functional, esthetic, and psychosocial benefits experienced by the patient, the PI provides insight into the biological conditions supporting the long‐term success of the prosthetic restoration.

Several prosthetic treatment options were considered for the presented patient. Implant‐supported FDPs are widely regarded as the treatment of choice for replacement of missing teeth because they preserve adjacent tooth structure, facilitate oral hygiene, maintain alveolar bone, and demonstrate excellent long‐term survival [20]. However, successful implant placement depends largely on adequate bone volume and favorable soft tissue architecture. Severe ridge deficiencies frequently require extensive augmentation procedures before implant placement. Vertical bone augmentation exceeding approximately 2.5 mm has been associated with an increased risk of biological and surgical complications [21]. In addition, implant therapy involves multiple surgical procedures, prolonged healing periods, increased treatment costs, and potential biological and mechanical complications, including peri‐implant disease, implant fracture, screw loosening, and prosthetic component failure [22–24]. In the present case, the combination of extensive vertical and horizontal bone loss, the need for major reconstructive surgery, and the uncertain long‐term prognosis of bone augmentation made implant‐supported rehabilitation a less predictable treatment option. Furthermore, the patient expressed a clear preference for a fixed restoration while avoiding additional surgical interventions. These factors ultimately led to the selection of a conventional tooth‐supported FDP.

RPDs remain a conservative and relatively inexpensive treatment alternative for patients with extensive ridge defects. However, removable prostheses are frequently associated with compromised retention and stability, mucosal irritation, impaired masticatory efficiency, and patient discomfort [25, 26]. Adaptation to removable prostheses may require a prolonged psychological adjustment period, and several studies have reported lower patient satisfaction compared with fixed prosthetic solutions [27, 28]. Considering the patient′s expectations for comfort, esthetics, and fixed rehabilitation, removable treatment was considered less favorable.

Conventional tooth‐supported FDPs continue to represent a reliable treatment option when implant therapy is contraindicated or declined by the patient [29]. Proper case selection remains essential, particularly in long‐span restorations where biomechanical considerations significantly influence long‐term success. The remaining abutment teeth must provide sufficient periodontal support and satisfy the principles described by Ante′s law, which recommends that the total periodontal ligament surface area of the abutment teeth should equal or exceed that of the teeth being replaced [30].

For this reason, the initial treatment plan was modified after evaluation of the provisional restoration. Although satisfactory function was achieved with premolars serving as abutments, both maxillary first molars were subsequently included to improve load distribution and increase the rigidity of the prosthesis. Expanding the number of abutments reduced the bending forces acting on the framework and improved the biomechanical prognosis of the restoration. The provisional restoration also provided valuable information regarding esthetics, phonetics, occlusion, and the patient′s ability to maintain satisfactory oral hygiene before fabrication of the definitive prosthesis. In the present case, the maxillary right second molar was considered to have an unfavorable long‐term prognosis and was therefore deliberately excluded from the definitive FDP, with the first molar serving as the terminal abutment on the right side. This approach ensured that the potential future loss of second molar would not directly compromise the structural integrity of the FDP or necessitate its removal. Nevertheless, extraction of second molar could reduce posterior occlusal support and result in a greater concentration of functional loads on the remaining dentition and the long‐span restoration. Therefore, regular clinical and radiographic monitoring of second molar, the FDP abutments, and the occlusal contacts is warranted. Should extraction of second molar become necessary, the occlusal scheme should be reassessed and adjusted accordingly. Subsequent rehabilitation of the mandibular arch was also planned to increase the number and improve the bilateral distribution of occlusal contacts, enhance posterior support, facilitate harmonious mandibular movements, and reduce mechanical loading on the maxillary FDP. However, because the prosthetic treatment was not covered by the patient′s health insurance and all treatment costs were borne by the patient, simultaneous rehabilitation of both arches was not financially feasible. Consequently, mandibular rehabilitation was postponed to a subsequent treatment phase.

Long‐span FDPs provide several important advantages, including excellent patient comfort, superior esthetics, restoration of phonetics, preservation of proprioception through natural abutment teeth, and elimination of palatal coverage [31–35]. Nevertheless, increasing prosthesis length inevitably increases the risk of framework flexure and technical complications [30, 35–37]. Connector dimensions, framework design, prosthesis configuration, and the number and distribution of abutment teeth are therefore critical determinants of long‐term success. The connector region has consistently been identified as the area of the highest stress concentration and the most common site of fracture initiation [38].

Material selection represented another important aspect of treatment planning. Although zirconia‐based restorations have become increasingly popular because of their favorable esthetics and biocompatibility, porcelain‐fused‐to‐metal restorations continue to demonstrate excellent mechanical performance and long‐term clinical reliability, particularly in extensive FDPs prostheses [39, 40]. The metallic framework effectively distributes functional stresses, reducing the risk of catastrophic fracture compared with all‐ceramic alternatives. Although the presence of a metal substructure may compromise optical properties, careful ceramic layering allows satisfactory esthetic outcomes, especially when combined with adequate prosthetic design [35, 39, 41].

Considering the size of the pontic segment required to restore the lost alveolar ridge, special attention was directed toward minimizing the overall weight of the prosthesis. A hollow metal framework was therefore designed and fabricated using additive manufacturing technology. This approach reduced the weight of the definitive restoration while maintaining sufficient rigidity to support the veneering ceramic. Although evidence regarding hollow metal frameworks in extensive prostheses remains limited, reducing prosthesis mass may decrease functional loading on the abutment teeth while improving patient comfort. The favorable clinical outcome observed in this case supports the potential usefulness of this design in carefully selected patients.

Regardless of the prosthetic treatment modality, long‐term success depends largely on meticulous plaque control and regular maintenance. Long‐span FDPs present additional challenges for oral hygiene because of their extensive pontic surfaces and connector regions. Studies have consistently demonstrated that patient compliance with oral hygiene recommendations improves significantly when comprehensive instructions are provided by the treating clinician [42]. In the present case, the patient maintained excellent oral hygiene throughout treatment and follow‐up, contributing to the favorable biological and functional outcome.

Within the limitations of a single case report, this clinical presentation demonstrates that a carefully planned long‐span porcelain‐fused‐to‐metal FDP with a hollow metal framework may represent a predictable treatment option for patients with severe Seibert Class III ridge defects who are not suitable candidates for implant therapy or decline surgical reconstruction. Careful patient selection, sound biomechanical planning, appropriate material selection, and strict maintenance protocols remain the key factors for achieving successful long‐term outcomes.

## 4. Conclusion

Long‐span porcelain‐fused‐to‐metal, tooth‐supported FDPs represent a reliable treatment option for patients with extensive anterior maxillary defect and missing teeth when implant therapy is not feasible. With careful case selection, appropriate prosthetic design, and meticulous treatment planning, satisfactory functional and esthetic outcomes can be achieved. Regular follow‐up and proper oral hygiene are essential for the long‐term success of this treatment approach.

## Author Contributions

**Aleksandra Prokić:** conceptualization, data curation, formal analysis, investigation, methodology, writing—original draft. **Stefan Vulović:** data curation, formal analysis, investigation, validation, writing—original draft. **David Krcunović:** data curation, funding acquisition, investigation, resources, writing—original draft. **Miloš Pelemiš:** formal analysis, investigation, methodology, visualization, writing—original draft. **Aleksandra Milić Lemić:** conceptualization, funding acquisition, methodology, project administration, resources, supervision, writing—review and editing.

## Funding

This work was supported by Republic of Serbia, Ministry of Education, Science and Technological Development (Grant Number: 451‐03‐34/2026‐03/200129; Belgrade 05.02.2026).

## Disclosure

All authors have read and approved the final version of the manuscript. The corresponding author had full access to all of the data in this study and takes complete responsibility for the integrity of the data and the accuracy of the data analysis.

## Ethics Statement

The study was conducted in accordance with the Declaration of Helsinki. This case report was prepared and reported in accordance with the CARE (CAse REport) reporting guidelines. As this report describes a single anonymized clinical case managed according to routine standards of care, formal ethical committee approval was not required.

## Consent

Written informed consent for treatment, clinical documentation, and publication was obtained from the patient.

## Conflicts of Interest

The authors declare no conflicts of interest.

## Supporting information


**Supporting Information** Additional supporting information can be found online in the Supporting Information section. File S1: CARE checklist.

## Data Availability

The data that support the findings of this study are available on request from the corresponding author. The data are not publicly available due to privacy or ethical restrictions.
